# Motion artefact removal in electroencephalography and electrocardiography by using multichannel inertial measurement units and adaptive filtering

**DOI:** 10.1049/htl2.12016

**Published:** 2021-06-24

**Authors:** Christopher Beach, Mingjie Li, Ertan Balaban, Alexander J. Casson

**Affiliations:** ^1^ Department of Electrical and Electronic Engineering The University of Manchester Manchester UK; ^2^ Design Engineer, Arm Ltd. Manchester UK

## Abstract

This paper presents a new active electrode design for electroencephalogram (EEG) and electrocardiogram (ECG) sensors based on inertial measurement units to remove motion artefacts during signal acquisition. Rather than measuring motion data from a single source for the entire recording unit, inertial measurement units are attached to each individual EEG or ECG electrode to collect local movement data. This data is then used to remove the motion artefact by using normalised least mean square adaptive filtering. Results show that the proposed active electrode design can reduce motion contamination from EEG and ECG signals in chest movement and head swinging motion scenarios. However, it is found that the performance varies, necessitating the need for the algorithm to be paired with more sophisticated signal processing to identify scenarios where it is beneficial in terms of improving signal quality. The new instrumentation hardware allows data driven artefact removal to be performed, providing a new data driven approach compared to widely used blind‐source separation methods, and helps enable in the wild EEG recordings to be performed.

## 1 INTRODUCTION

Electroencephalography (EEG) and electrocardiography (ECG) are commonly used bio‐sensing approaches which place electrodes on the head and chest respectively to record the electrical activity from within the body. This information is of use in a wide range of clinical applications, from atrial fibrillation detection [1] to epilepsy diagnosis [2], and in non‐clinical applications such as Brain‐Computer Interfaces [3]. Wearable EEG and ECG devices provide convenient and inexpensive methods for monitoring a subject's brain and heart in a miniaturised and portable way, and the devices are finding many applications in preventative healthcare [4].

However, being microvolt level signals, the EEG and ECG are very susceptible to environmental interference such as mechanical disturbances which deteriorate the electrode coupling to the user's body. Motion artefacts due to body movement during signal acquisition have been a great obstacle for traditional EEG and ECG performed out‐of‐the‐lab [2, 4]. In general, wearable monitoring of the ECG is possible as the ECG waveform is large compared to the EEG, and a number of commercial patch‐like devices are available, for example the VitalPatch® [5]. Nevertheless, ECG wearables are still subject to motion interference, necessitating specialised algorithms for cleaning the data and detecting each heart beat [6]. Robustness to motion artefacts is essential for heart rate variability applications of ECG data [7], where the incorrect detection of just two heart beats can lead to incorrect heart rate variability measures being obtained [8]. For EEG, out‐of‐the‐lab portable monitoring is only just starting to become available [9, 10], and is still very challenging due to the motion artefacts that are collected together with the wanted EEG signal. As a result there has been a significant amount of interest in methods for removing artefacts from electrophysiological signals in recent years [11].

Recent methods for motion artefact removal, particularly for EEG, are mainly based on blind source separation (BSS) approaches such as independent component analysis (ICA) [12]. These methods are popular and widely used, but require a large number of recording channels and high computational power for good performance. They are thus not suitable for wearable applications which aim to have a low number of parallel channels in order to be quick to set up, be small and socially discrete, and where the available processing power is limited by power consumption constraints. In addition, BSS methods are blind to the wider sensing situation present, i.e. they do not make use of additional meta‐signals to help with motion artefact removal. Compared to BSS approaches, works on data driven motion artefact removal are much more limited. Previous works have taken continuous measurements of the impedance at the electrode‐skin interface in EEG recordings to use as an input to an adaptive filter [13, 14]. However, taking a continuous impedance measurement during bio‐signal acquisition does not provide a direct measure of the motion present, and requires injecting a current into the body which needs a good electrode contact.

In contrast, direct recordings of motion are now easily possible with the widespread availability of inertial measurement units (IMUs) containing accelerometers, gyroscopes and magnetometers [15]. IMUs are now widely used in fitness trackers, but their data is not routinely used for removing motion artefacts from electrophysiological signals. For ECG, [16] used IMUs to estimate the local electrode motion which was used as the reference signal for baseline wander reduction. For EEG, while accelerometers are common in many EEG recording units, usually only one is included for the entire system, which implies that the motion effects on each channel are assumed to be equal [17, 18, 19]. A per‐channel adaptive filter for the removal of the local motion artefact is thus not possible.

To tackle this problem, we designed and implemented a multichannel EEG and ECG device with IMUs on each active sensing electrode and verified the feasibility of combining each electrode motion data and the EEG/ECG signal data to achieve motion artefact removal. To our knowledge this is the first application of IMU driven adaptive filtering for the removal of motion artefacts from EEG, allowing data driven motion artefact removal. In this paper Section 2 describes the hardware design of our new active electrodes and Section 3 details the adaptive filtering technique and experimental setup. Our validation methods are given in Section 4 with performance results of the IMU driven motion artefact removal given in Section 5. Conclusions drawn in Section 6.

## 2 HARDWARE AND FIRMWARE DESIGN

**FIGURE 1:**
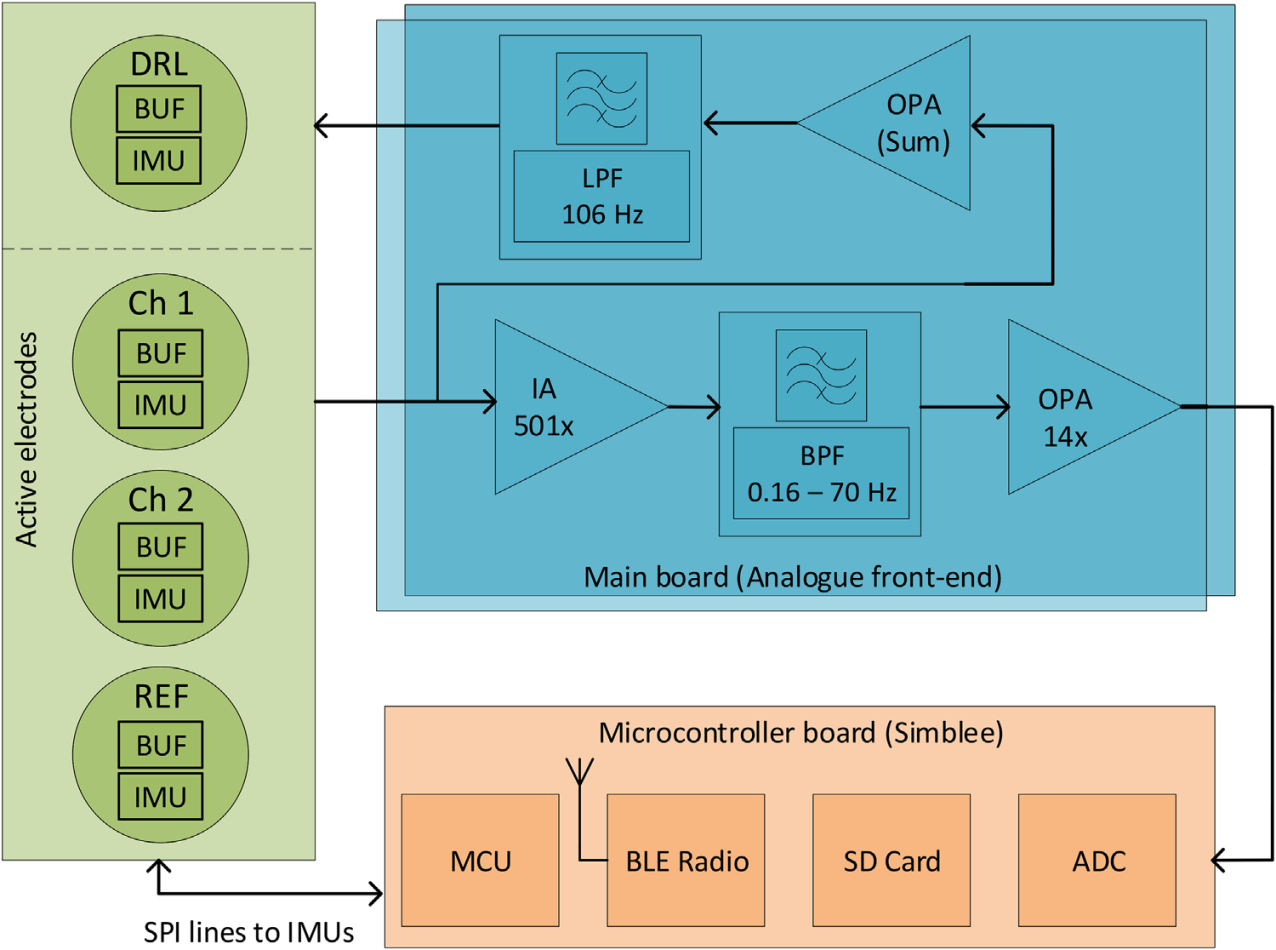
Proposed overall design architecture. BUF = buffer amplifier, IA = instrumentation amplifier, all other acronyms are defined in the text

Figure 1 shows our overall system architecture. The device consists of three subsections: active electrodes incorporating IMUs and a bio‐potential amplifier; a main board processing the analogue signals; and a microcontroller board controlling the conditioning circuitry and collecting and storing the EEG/ECG signal data. All sections are powered by a single battery.

For EEG/ECG collection our active electrode design is based upon that from [20]. The input channels of our design have a unipolar EEG/ECG measurement strategy, with two active channels, one shared reference channel (REF), and one driven right leg (DRL) channel. The reference and active channel electrodes have identical circuitry. The DRL electrode has the same shape as the active and reference channel electrodes but the circuit is different, consisting of the body driving electronics.

Each active electrode consists of a unity gain precision dual channel low power operational amplifier (OPA) (Linear Technology LTC6078) used for buffering on the electrode board. On the main processing board the two active channel signals are pre‐amplified by an Instrumentation Amplifier (IA) (Texas Instruments INA128) with a gain of 501. Pre‐amplified signals then go through a bandpass Butterworth filter (BPF) on the main board, built around an LTC6078 OPA. This circuit block has a high pass filter (HPF) with frequency cut‐off of 0.16 Hz, implemented as a passive RC filter, followed by a first order active non‐inverting low pass filter (LPF) with frequency cut‐off of 70 Hz and an amplification of 14. The filtered signal is selected through an analogue multiplexer and sampled by the 10‐bit built‐in analogue‐to‐digital converter (ADC) of an Arm Cortex‐M0 microcontroller (MCU) board (Simblee RFD77101 [21]). The gathered channels and reference signal are summed and averaged, then fed back into the body through the DRL channel with inversion (inverting LPF with cut‐off 106 Hz built around a LTC6078 OPA) to improve the common mode rejection ratio (CMRR) as described in [22].

In addition to this circuitry, each individual active electrode has a miniature low power three‐axis accelerometer and gyroscope IMU (STMicroelectronics LSM6DS3 [23]) mounted on it. We opted not to use a magnetometer in this system to reduce power consumption and due to these sensors being very sensitive to local magnetic field disruptions [24]. Also, previous work with optical sensors has shown that adequate motion artefact removal can be undertaken with just an accelerometer and gyroscope [25]. The motion of each electrode is acquired by these IMUs, and transferred to the microcontroller through the serial peripheral interface (SPI). All of the acquired electrophysiological data and motion data are then stored on an SD card by the microcontroller. Collected data are loaded to a personal computer for processing offline. Un‐used analogue channels of the multiplexer are used as demultiplexer channels for the digital control of the chip select of the IMUs. A wireless BLE (bluetooth low energy) radio is present for real‐time streaming of data, but turned off by default as it is not possible to stream all of the bio‐potential and IMU data within the bandwidth of the BLE link on the Simblee device.

**FIGURE 2:**
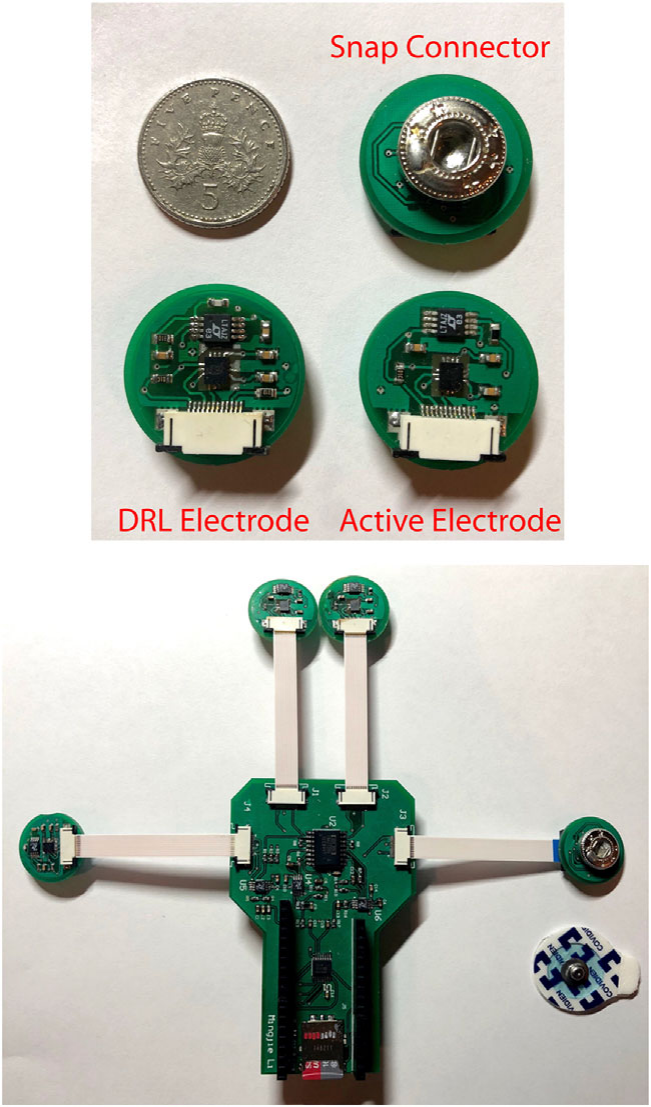
Photograph of the fabricated device. (Top) PCB design of DRL and active electrode compared to a UK 5p. (Bottom) The electronic circuit used to record EEG and ECG signals together with a standard self‐adhesive ECG electrode

Our manufactured device is shown in Figure 2. The active electrode PCB design (Figure 2(a)) has a circular shape with a weight of 1.7 g and size of 18 mm in diameter. To balance the electrode, and remove additional electrode movement due to the weight of the IMU, the weight of the components is distributed as evenly as possible on the PCB, and the IMU is placed at the centre of the electrode PCB on the top layer. A snap connector is placed at the centre on the bottom layer to allow the proposed electrode to plug in to standard both wet and dry electrodes [26]. Figure 2(b) shows the overall circuit consisting of 4 electrodes attached to a main board with FFC connectors, the microcontroller, and SD card.

The board is set to sample all signals at 220 Hz, with a 0.5 μV least significant bit (LSB) to allow the digitisation of both ECG and EEG signals in‐line with clinical EEG recommendations [27]. The range of the accelerometer is set as ±16 g with a linear acceleration sensitivity of 0.488 mg/LSB, and the gyroscope is set to a range of ±2000 dps with an angular rate sensitivity of 70 mdps/LSB. Both have 16 bit sampling.

## 3 ADAPTIVE FILTERING FOR MOTION ARTEFACT REMOVAL

Adaptive filtering was performed offline on a PC (MATLAB R2020a), utilising the data recorded on the SD card. All recorded bio‐signals were first filtered by Butterworth fourth‐order zero‐phase bandpass filters (0.16–40 Hz for EEG, 0.05–100 Hz for ECG), followed by filtering with a fourth‐order zero‐phase notch filter (47.5–52.5 Hz) to remove mains‐line noise. Initial analysis identified that motion artefacts on the bio‐signals were better correlated with velocity, rather than the acceleration signal recorded by the accelerometers. To account for this, we integrated the acceleration signal using cumulative trapezoidal numerical integration to generate an estimate of the velocity of each of the electrodes. The angular rotation data from the gyroscope was filtered with a third order Savitzky–Golay filter with a frame length of 51 to remove high‐frequency noise.

**FIGURE 3:**
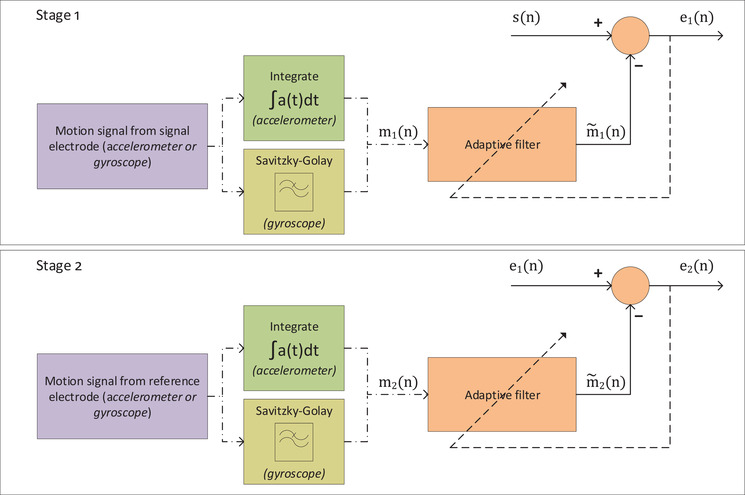
Diagram of the overall adaptive filtering approach demonstrating the two adaptive filter processing stages

To correct for drift between sensors, as well as phase delay between the physical movement of the person/electrode and the artefact manifesting on the bio‐signal, cross correlation between the bio‐signals and the IMU signals (the velocity and the angular rotation data) was calculated, up to a maximum shift of 330 samples. The respective IMU signals were shifted by the amount where correlation was highest. In this work the IMU signals from the accelerometer and gyroscope were not fused, instead we compare two different adaptive filters, one using the accelerometer signal as the motion estimate and the other using the gyroscope data as the motion estimate. In addition, rather than obtaining a composite signal of the motion from each IMU sensor by calculating the vector magnitude (which would remove the direction of the artefact), we only process a single axis of data from each IMU. In this work we select the axis which has the highest correlation with the bio‐signal data. The adaptive filtering processing steps are summarised in Figure 3 and detailed as follows.

The motion reference (from either the gyroscope or accelerometer) is used as one input to an adaptive filter, with the other input being the recorded bio‐signal, which consists of a mixture of the clean EEG/ECG trace and the motion artefact. To perform the adaptive filtering process, the measured bio‐signal is modelled as:

(1) $$s\left(n\right)=s_{\text{bio}}\left(n\right)+\tilde{m_{1}}\left(n\right)+\tilde{m_{2}}\left(n\right)+q\left(n\right),$$

where $s_{\text{bio}}\left(n\right)$ is the clean bio‐signal at sample times n, $\tilde{m_{1}}\left(n\right)$ is the motion artefact at the signal electrode, $\tilde{m_{2}}\left(n\right)$ the motion artefact at the reference electrode, and q(n) is the additional noise introduced by the overall circuit. Note that Equation (1) does not contain a motion artefact signal corresponding to the motion of the DRL electrode. As this electrode is driving the body rather than being a recording site, any noise injected by this electrode will be common to all recording electrodes and therefore attenuated by the common mode rejection of the amplifiers in the analogue front‐end circuitry.

As motion artefacts can manifest at either one or multiple electrodes, we model the motion artefacts as two separate components, and perform two stages of adaptive filtering in series to remove them, similar to the approach taken in [28] for removing EMG artefacts. The first stage is a normalised least mean square (NLMS) adaptive filter which filters the measured signal (contaminated bio‐signal) such that the output of the filter should be as close as possible to the first motion signal (captured by the IMU on the signal electrode), and the error is then the partially motion free bio‐signal (still corrupted with the motion artefacts from the reference electrode). This output error from the first stage adaptive filter is then fed into a second NLMS adaptive filter which is set so the output of the filter follows the reference electrode motion signal as closely as possible, and the error output is the fully motion free bio‐signal.

The motion artefacts for the two stages of the adaptive filter are modelled as:

(2) $$\tilde{m_{1}}\left(n\right)={w_{1}}^{T}\left(n\right)m_{1}\left(n\right)$$

and

(3) $$\tilde{m_{2}}\left(n\right)={w_{2}}^{T}\left(n\right)m_{2}\left(n\right)$$

where $m_{1}$ and $m_{2}$ are movement data from the signal and reference electrode respectively, being either the accelerometer or the gyroscope data depending on configuration. $\tilde{m_{1}}$ and $\tilde{m_{2}}$ are the estimated electrophysiological motion artefact resulting from the motion. The bold font denotes a vector, and w is the adaptively updated weight coefficients of the NLMS algorithm which should be updated by the error

(4) $$e_{1}\left(n\right)=s_{\text{bio}}\left(n\right)-\tilde{m_{1}}\left(n\right)$$

(5) $$e_{2}\left(n\right)=e_{1}\left(n\right)-\tilde{m_{2}}\left(n\right)$$

for the first and second stages of the adaptive filter. The weights w for both of the adaptive filters are updated by the following equation for an NLMS algorithm [29],

(6) $$w\left(n+1\right)=w\left(n\right)+\frac{\mu }{\varepsilon +∥m_{i}{\left(n\right)∥}^{2}}m_{i}\left(n\right)e\left(n\right)$$

where $m_{i}\left(n\right)$ is the motion signal (from either the accelerometer or the gyroscope depending which is selected) in either the first stage (i.e. i = 1), or second stage (i = 2). μ is the update step size, and ε is a parameter added for instability prevention if $∥m_{i}{\left(n\right)∥}^{2}$ is too small. In this work the filter length was set to 9 and the step size μ to 0.1.

## 4 VALIDATION AND VERIFICATION METHODS

The new ECG/EEG instrumentation system was verified by placing the unit on a volunteer and recording example signals. All procedures were approved by the University of Manchester Research Ethics committee, application 2019‐6137‐9413, and participants gave written informed consent before taking part.

For ECG, the proposed design was tested by placing the 4 electrodes in typical 12 lead ECG electrode locations [30]. The DRL electrode was placed on RL, the REF electrode on LL, and active channel electrodes 1 and 2 were placed on V1 and V2 respectively, using standard self‐adhesive Ag/AgCl pre‐gelled sensing pads. Motion artefacts were generated by marching on the spot and then by turning around.

For EEG, active channel electrodes 1 and 2 were placed either on T3 and T5 of the 10–20 system [2], near the left ear, or on T4 and T6 near the right ear. The DRL and REF were placed on Fp1 and Fp2 respectively. The electrodes were the same as those used for the ECG measurement. Data was collected with the head still and with the head swinging from side to side.

To verify the operation of the design we investigate the output of the adaptive filtering scheme with the accelerometer and the gyroscope respectively set as the input. For ECG data we run a Pan–Tompkins algorithm for R peak detection [31] on the data, to compare the performance of beat detection prior to and after motion artefact removal. We also measure the signal‐to‐noise ratio (SNR) of the unfiltered ECG waveform using our previously reported method [32]. Briefly, this comprises of calculating the SNR in each heart beat interval by taking the amplitude of the R‐peak as the signal and the root‐mean‐square of a section of the data between successive R‐peaks as the noise (a window of time from 1/2 to 3/4 between successive peaks). In addition, bench measurements of the device noise and sampling time were taken to investigate the practicality and scale‐ability of the new instrumentation design.

## 5 RESULTS AND DISCUSSION

The new instrumentation electronics operate from a 3.2 V supply, powered by two rechargeable AA batteries. The measured input‐referred noise is 2.3 μVrms, comparable to other EEG amplifiers such as the mBrainTrain Smarting Mobi [33]. Collecting each individual sample of a bio‐signal and IMU data takes 142 μs, allowing the circuitry to in principle scale to 32 simultaneous channels for a 220 Hz sample rate. In terms of power consumption, the LSM6DS3 IMU consumes a nominal 0.9 mA, 2.8 mW with the 3.2 V supply. In contrast each bio‐potential front‐end has a measured power consumption of 1 mA. In terms of direct sensing the power consumption is approximately doubled by the addition of the new motion sensing. However, much larger is the impact on the data storage/transmission with 7 data streams from each electrode (1 bio‐potential, 3 accelerometer, 3 gyroscope). The raw bio‐signal data rate is 2,200 bps, while from the IMU it is 21,120 bps, reducing battery life by a factor of approximately 10 [34]. In practical use a trade‐off between battery lifetime, miniaturisation/size of batteries, and the potential for improved data quality from the incorporation of IMUs is thus required.

**FIGURE 4:**
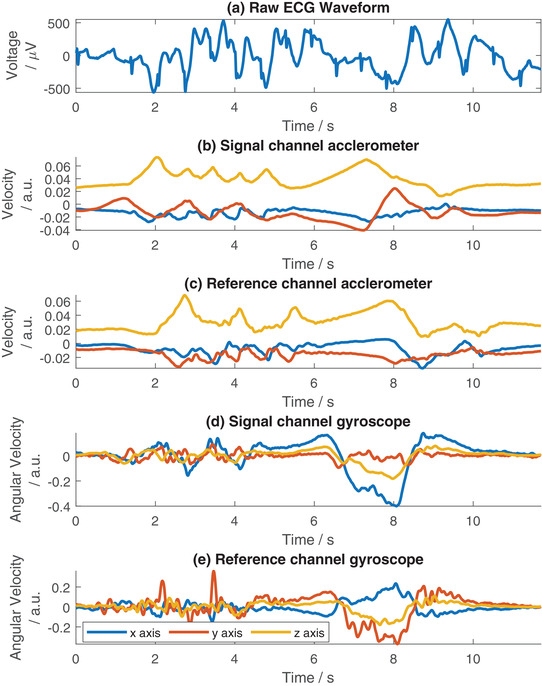
Example ECG recorded from V2 electrode and corresponding accelerometer and gyroscope data

In Figure 4 example signals from the device are shown. Here Figure 4(a) demonstrates the raw ECG signal collected from the V2 electrode. (Similar results are obtained for the V1 electrode, not included for space.) Clear QRS complexes are visible in the raw data, combined with motion artefacts which primarily manifest as low frequency shifts. The representation of the motion as recorded by the accelerometer (integrated to obtain the velocity) is shown in Figure 4(b, c) for the signal and reference electrodes respectively. The representation of the motion as recorded by the gyroscope (after Savitzky–Golay filtering) is shown in Figure 4(d, e). Visually it can be seen that the sudden motion artefacts around 2–5 s manifest in the accelerometer waveform as negatively correlated peaks. Comparing Figure 4(b) with Figure 4(c), in this 2–5 s window, it can be seen that more peaks relating to the motion are collected on the signal electrode than the reference electrode. The gyroscope data in Figure 4(d, e) does not show this as clearly, where the noise floor is higher and the specific artefacts are harder to discern by eye. At 5–9 s in the waveform, a slow motion artefact can be seen. Again this can be seen in both the accelerometer and gyroscope traces. However, the artefact is better correlated with the gyroscope data than the accelerometer. The x and y‐axes of the gyroscope data in Figure 4(d) show a similar trend to the artefact, whereas the accelerometer data in Figure 4(b) shows a trend in the waveform but some of the features, such as the switch in direction, are not closely correlated in time and the overall representation of the artefact takes longer to stabilise than in the bio‐signal.

**FIGURE 5:**
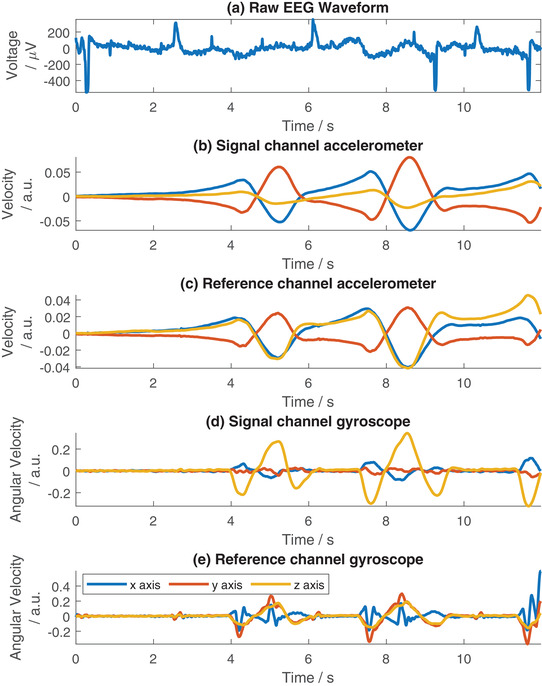
Example EEG recorded from T5 and corresponding accelerometer and gyroscope data

In Figure 5 we demonstrate example signals for the EEG waveform during eyes closed head swinging. Figure 5(a) shows the raw EEG signal. While it is more challenging to discern the desired underlying signal here as there are not clear features to identify like the QRS complex in an ECG waveform, we can identify certain features such as eye blinks. These can be identified at 0.8, 2.2, 3.5, 4.8, 5.6, 8.7 and 10.0 s as low amplitude sharp deflections, as well as eye saccades (movements) which can be seen at 0.3, 2.6, 6.1, 9.2, 10.3 and 11.7 s. These eye movements can be used as reference features to identify the performance of the EEG adaptive filtering. Figure 5(b, c) shows the accelerometer data from the signal and reference electrode respectively, while Figure 5(d, e) show the gyroscope data from the signal and reference electrode respectively. The head swinging motion artefacts can be identified in each of the motion signals and can be seen overlaid on the EEG waveform in Figure 5(a) as slow baseline shifts.

**FIGURE 6:**
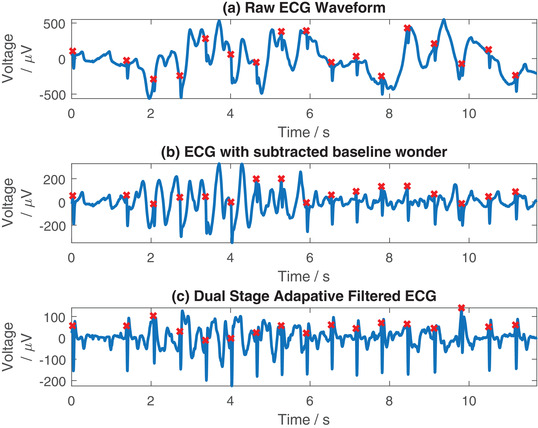
Example comparing filtering of data using wavelet baseline removal (b) against the adaptive filtering method proposed in this paper (c). Red crosses indicate identified QRS complexes from the Pan–Tompkins algorithm

In Figure 6 we compare the raw ECG waveform in Figure 6(a) against two filtering methods: the removal of the baseline wander using a discrete wavelet transform as described in [35] shown in Figure 6(b); and filtering using the adaptive filtering with the accelerometer data in Figure 6(c). All subplots in this figure highlight identified QRS complexes by the Pan–Tompkins algorithm, indicated by red crosses. Here we can identify the performance benefit of using this adaptive filter over simple baseline removal. The signal in Figure 6(c) shows more clearly identifiable morphological features (such as the P and T‐waves) than the method based purely on baseline filtering alone in Figure 6(b). This demonstrates that even though the artefacts manifest as baseline shifts, attempting to remove them using baseline removal does not sufficiently remove the artefacts. Despite each of the subplots containing artefacts, the Pan–Tompkins algorithm is able to correctly identify all of the QRS complexes, even prior to the adaptive filtering. The advantage of using the filtering is to clean up the record to allow identification of the ECG morphology, although in cases where the motion artefacts are more substantial than in this example, it is possible that the Pan–Tompkins algorithm would only be able to perform adequately on the filtered data.

**FIGURE 7:**
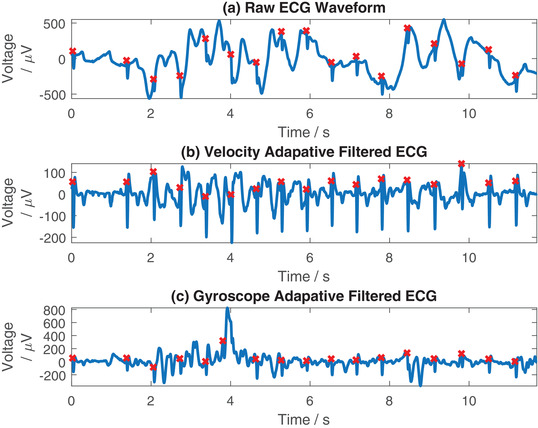
Example of a good case, filtering ECG using the accelerometer and gyroscope independently

**FIGURE 8:**
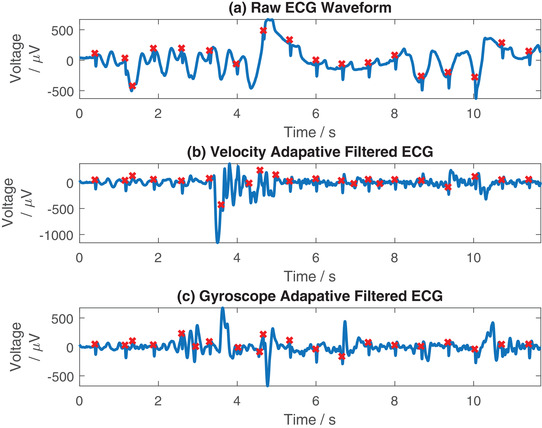
Example of a poor case, filtering ECG using the accelerometer and gyroscope independently

In Figure 7, we demonstrate filtering of an ECG record comparing the performance of the accelerometer and gyroscope based adaptive filtering configurations. The unfiltered data is shown in Figure 7(a), with the data filtered using the accelerometer data in Figure 7(b), and the data filtered using the gyroscope data in Figure 7(c). Visually, filtering with the accelerometer data has better performance than filtering with the gyroscope data, with the gyroscope based filtering introducing extra artefacts into the signal around the 2–4 s period. However, if we compare a section of the data later on in the record, at 6–8 s, where there is a large slow motion artefact in the data, the gyroscope filtered data provides good performance. Here, the gyroscope is more sensitive to sudden motion artefacts, and is better synchronised with the artefact at 6–8 s than the accelerometer, therefore it generates better adaptive filtering performance. In Figure 8(a) we demonstrate the filtering of another ECG record corrupted with motion artefacts, filtering using the accelerometer data as the input in Figure 8(b) and the gyroscope as the input in Figure 8(c). Again, we have performed Pan–Tompkins beat detection on each to automatically identify QRS complexes. Here, it can be seen that the filtering process has performed considerably worse than in Figure 7, adding additional noise to the ECG waveform as well as reducing the performance of the Pan–Tompkins algorithm, causing it to detect additional QRS complexes that were previously accurately determined in the raw waveform. Calculating the SNR of the raw waveforms in both Figures 7(a) and 8(a), these are 18.14 and 21.76 dB respectively. In the case of the data presented here, we find that the adaptive filtering is beneficial in the lower SNR signal, while in the higher SNR ECG signal we find that the adaptive filtering can reduce the quality of the signal rather than improving it. This suggests that future practical implementations of this setup may require a method of automatically switching between the adaptive filtered data and the unfiltered data, and between accelerometer and gyroscope inputs, based upon the SNR in order to create the cleanest output signal. However, more data collection is required to identify if this will be beneficial.

**FIGURE 9:**
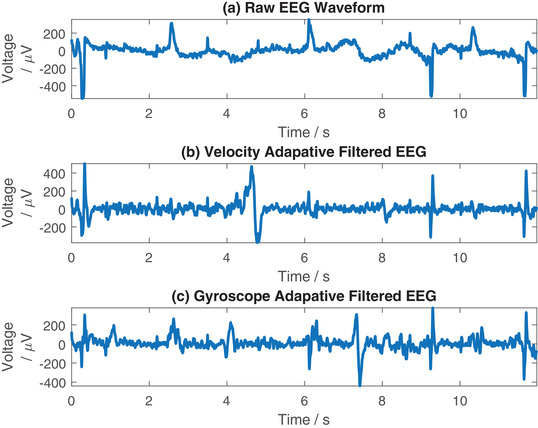
Example filtering EEG using the accelerometer and gyroscope independently

Figure 9 shows an example of adaptive filtering applied to EEG data, with the raw data in Figure 9(a), the data filtered with the accelerometer shown in Figure 9(b), and the data filtered with the gyroscope in Figure 9(c). Here, we use the eye movement artefacts (blinks and saccades) as a reference for an underlying signal. As previously discussed it is more challenging with an EEG waveform to identify successful filtering as the desired underlying signal is unknown. However, some common features can be identified in the filtered signals in both records, such as eye blinks, which are largely still present in Figure 9(b, c) at 0.8, 2.2, 3.5, 4.8, 5.6, 8.7 and 10.0 s, although with a lower noise floor. The eye saccade artefacts at 0.3, 2.6, 6.1, 9.2, 10.3 and 11.7 s are also still present in both filtered signals, again with a change in magnitude. The slow baseline wander that is present in the unfiltered waveform in Figure 9(a) created from the head shaking has been removed in Figure 9(b, c), although the noise floor of the signal has been increased. The filtering process has also introduced additional artefacts, at 4.7 s in Figure 9(b) and at 7.4 s in Figure 9(c) at 7.4 s. Given the increase in noise floor and introduction of additional artefacts, we deduce that with the limited data collected in this paper the benefit of using our adaptive filtering process for EEG records is not clear and more sophisticated processing may be required to use motion‐based adaptive filtering with EEG signals. Nevertheless, our system allows us to demonstrate the principle of data driven artefact removal for EEG systems, which can be built upon in future works to improve performance.

## 6 CONCLUSION

This paper has proposed an EEG and ECG acquisition device with an individual IMU implemented on each active electrode for motion artefact removal by adaptive filtering. Although implementing individual IMUs on each electrode increases the power consumption and the data acquisition load during sampling, it enables data driven removal of the motion artefact. The results suggest that this adaptive filtering performs better motion artefact removal when the raw signal has a lower SNR and performs more poorly with a higher SNR input signal, although more data collection is required to prove this is the case. We also identify that the processing does not perform optimally on the EEG data collected in this paper.

Further, we identify that the gyroscope is more sensitive to motion artefacts which can provide better filtering performance where it is better correlated with the motion artefact, but can also introduce additional noise into the filtered signal. For these reasons, it is recommended that our adaptive filtering algorithms are paired with a process to automatically switch between the filtered and unfiltered records depending on which gives a higher quality signal. Future work should focus on an online implementation of the artefact removal approach, and characterising the EEG and ECG artefacts compared to the recorded motion of the actual electrode, which our hardware approach enables for the first time. Further, multi‐channel adaptive filters as in [36] should be investigated to allow the multiple input streams (multiple axes of both accelerometer and gyroscope data) to be fused and the best combination chosen automatically, which may lead to better performance than the cascaded filters used here.

## FUNDING AND DECLARATION OF INTERESTS

This work was supported in part by the UK Engineering and Physical Sciences Research Council grant numbers EP/P02713X/1 and EP/S020179/1.

## Data Availability

The data that supports the findings of this study are openly available in figshare at https://doi.org/10.48420/13626395.
